# The Electrochemical Stability of Starch Carbon as an Important Property in the Construction of a Lithium-Ion Cell

**DOI:** 10.3390/e23070861

**Published:** 2021-07-05

**Authors:** Beata Kurc, Marita Pigłowska, Łukasz Rymaniak

**Affiliations:** 1 Institute of Chemistry and Electrochemistry, Faculty of Chemical Technology, Poznan University of Technology, Berdychowo 4, PL-60965 Poznan, Poland; 2 Faculty of Chemical Technology, Poznan University of Technology, Berdychowo 4, PL-60965 Poznan, Poland; marita.piglowska@student.put.poznan.pl; 3 Institute of Combustion Engines and Powertrains, Faculty of Civil and Transport Engineering, Poznan University of Technology, Piotrowo 3, PL-60965 Poznan, Poland; lukasz.rymaniak@put.poznan.pl

**Keywords:** starch, carbon, EIS, Bode plots, SEI

## Abstract

This paper shows use of starch-based carbon (CSC) and graphene as the anode electrode for lithium-ion cell. To describe electrochemical stability of the half-cell system and kinetic parameters of charging process in different temperatures, electrochemical impedance spectroscopy (EIS) measurement was adopted. It has been shown that smaller resistances are observed for CSC. Additionally, Bode plots show high electrochemical stability at higher temperatures. The activation energy for the SEI (solid–electrolyte interface) layer, charge transfer, and electrolyte were in the ranges of 24.06–25.33, 68.18–118.55, and 13.84–15.22 kJ mol^−1^, respectively. Moreover, the activation energy of most processes is smaller for CSC, which means that this electrode could serve as an eco-friendly biodegradable lithium-ion cell element.

## 1. Introduction

Currently, one research subject of interest to scientists is the use of starch-containing raw materials. It is a biopolymer saccharide, referred to as green, due to its high biodegradability. It occurs naturally as an energy store, including in leaves, rice, potatoes, wheat, peas, bananas, corn, and tapioca. When this biopolymer is used in electrochemistry, the following properties of starch are used: availability, renewable, biodegradability, non-toxicity, stability, and low price [1,2,3]. Swelling of granules, gelling, or retrogradation are important processes that allow the use of starch in the appropriate form as electrode material in lithium-ion cells. This enables a multiple increase in the stability of the capacity and the Coulombic efficiency of the cell [4,5,6]. Anode materials determine the storage capacity of the cells. Amylopectin chains show the same spatial structure as graphite, commonly used as an anode [7]. A significant role is played by the possibility of reversible change of its form from liquid to solid under appropriate conditions. A material with this ability is known as a non-Newtonian liquid (pseudoplastic material). This means that the material shows elastic and sticky features, which increases the safety of the cell’s operation. In order to use starch as an organic anodic active material, difficulties such as low Coulombic efficiency, large increase in volume, loss of capacity, and poor ion transport must be overcome [8,9,10,11].

**Figure 1:**
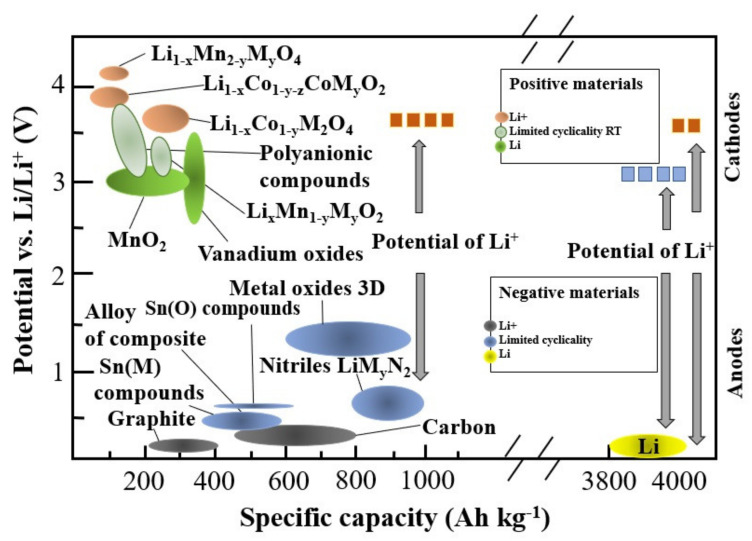
Anode materials using in Li-ion batteries (LIBs) [9,12,13].

Figure 1 shows a comparison of potential capabilities and capacitive anode and cathode materials. The highest specific capacity is shown by lithium, which is not commonly used due to the increased explosiveness of a cell constructed in this way and the formation of dendrites. The classic anode solution turns out to be graphite, which, however, has a limited capacity (372 mAh g^−1^). It is replaced with other carbon materials, including graphene as well as nanocomposite materials. Tin compounds and metal oxides are also used as anodes.

Dynamic frequency characteristics are part of the so-called frequency analysis of signals. The frequency characteristics belong to the dynamic group. They determine the behavior of the system in a sinusoidal steady state. If a sinusoidal signal is introduced to the input of linear and stationary systems, then after the expiration of the transients, a sinusoidal signal of the same frequency will also appear in the output. In general, the output signal has a different amplitude to the input signal and lags in phase. The system can be fully described using the given behavior, namely by presenting the ratio of the output amplitude to the input amplitude and the phase difference over the entire frequency range from zero to infinity. The frequency characteristics can be taken experimentally, and on their basis, it is possible to identify the dynamic properties of the processes. Due to the unique relationship between the graphic form of the description of processes expressed by frequency characteristics and the analytical form, in the operator form, by knowing the latter form, one can plot the frequency characteristics of any process.

The method of electrochemical impedance spectroscopy (EIS) consists in measuring the impedance value between the tested electrode (polarized to a specific potential in relation to the reference electrode) and the auxiliary electrode in the widest possible frequency range from several hundred kHz to 10^−3^ Hz. EIS is used to determine the speed of electrode reactions, the electrode processes taking place, and the characteristics of the electrode–electrolyte solution interface. It also allows the indirect assessment of the structure of the electrode surface [14,15,16,17,18].

In the case of a quasi-reversible reaction, the speed of the charge transfer process is similar to the mass transport speed. The reaction is controlled by charge transfer at high frequencies and diffusion at low frequencies. The total electrode impedance for this case consists of the solution resistance *R_S_*, the capacitance of the electric double layer *C_dl_*, and the Faraday impedance *Z_f_,* which in turn consists of the charge transfer resistance (activation resistance) *R_ct_* of the Warburg impedance *Z_w_* related to the transport of the reactant (diffusion). The equivalent electrical circuit for this system was presented by Randles. The total impedance for this circuit is:

$$Z\left(j\omega \right)=R_{s}+\frac{R_{ct}+Z_{w}}{1+j\omega C_{dl}\left(R_{ct}+Z_{w}\right)}$$

In the case when the process of charge transfer through the electrolyte solution–electrode interface is slower than the mass transport, the reaction that occurs is irreversible. The speed of this reaction is related to the rate of charge transfer; in this case, the Warburg impedance can be neglected. The total electrode impedance consists of the solution resistance *R_S_*, the electrical double layer capacitance *C_dl_*, and the charge transfer resistance (activation resistance) *R_ct_*. The equivalent electrical model consists of a series connection of the resistance *R_S_* with a parallel circuit composed of the capacitance *C_dl_* and the resistance *R_ct_*.

The equivalent electrical model consists of a series connection of the resistance *R_S_* with a parallel circuit composed of the capacitance *C_dl_* and the resistance *R_ct_*. The total impedance of this circuit is:

$$Z\left(j\omega \right)=R_{s}+\frac{1}{\frac{1}{R_{ct}}+j\omega C_{dl}}=R_{s}+\frac{R_{ct}}{1+j\omega R_{ct}C_{dl}}=R_{s}+\frac{R_{ct}}{1+\omega^{2}R_{ct}^{2}C_{dl}^{2}}-\frac{j\omega^{2}C_{dl}}{1+\omega^{2}R_{ct}^{2}C_{dl}^{2}}$$

In the case of a reversible electrode reaction, the process of charge transfer across the electrolyte solution–electrode interface is faster than mass transfer. The speed of this reaction depends on the diffusion rate, as it is the slowest reaction step. In this case, the charge transfer impedance can be neglected. The equivalent electrical model consists of a series connection of the resistance *R_S_* with a parallel circuit composed of the capacitance *C_dl_* from the Warburg impedance (diffusion impedance) *Z_w_*, conditioned by the diffusion transport of the reactants and products. The total impedance of this circuit is:

$$Z\left(j\omega \right)=R_{s}+\frac{Z_{w}}{1+j\omega C_{dl}Z_{w}}$$

In the literature, we find that the pseudo-capacity behavior and conductivity of, for example, manganese oxide are still not fully understood [19]. Some research groups have attempted to test this material [20,21,22] by electrochemical impedance spectroscopy (EIS). Thanks to EIS, it is possible to obtain information on the processes of charge transfer and diffusion, as well as on the loading of the double layer and the active layer of the material, which is not possible in the case of cyclic voltammetry (CV) [23,24,25,26,27,28,29,30,31,32].

Nowadays, the demand for energy around the world is increasing significantly. It is important to develop modern technologies of obtaining electricity and electrical installations in order to store the obtained energy. Currently, one of the best solutions is lithium-ion batteries. However, there is still an urgent need to design and manufacture lithium-ion cells with higher capacity, energy density, increased safety, and longer service life. Classic, commercial lithium-ion technology based on a lithium oxide cathode, such as LiNi1-x-yCo_x_Mn_y_O_2_, and a graphite anode has reached its theoretical limits. Among alternative anode materials, nano-structured metal oxides have been proposed. While high capacity has been achieved for the various compounds tested, many serious problems (especially the significant loss of capacity during battery use) remain unresolved. The materials presented in the paper, in the next stage of the research, were synthesized in order to obtain oxides with high entropy; they turned out to be excellent candidates to overcome these limitations. 

The definitions of these compounds are based solely on compositional foundations, without referring to specific thermodynamic issues, which in later studies strongly influenced the perception of high-entropic alloys [33,34,35,36,37,38,39,40,41,42]. Despite the differences in defining high entropy alloys, all novice researchers noticed the effect characteristic of these materials, which were first described and named by Yeh [37,38]. Yeh noted that these materials have specific significant effects, which are considered “core effects”, unnoticeable or poorly visible in traditional metallic (dominant element) materials. These effects are the effect of high entropy, the effect of significant distortion of the lattice, the effect of slow diffusion, and a synergistic effect. High entropy effect; conventionally, traditional alloys with one dominant element, including steel, nickel, aluminum, or titanium alloys, are characterized by the possibility of the formation of many phases, i.e., chemically and physically homogeneous elements of the system, which results directly from the Gibbs phase rule. According to the assumptions resulting from this rule, in multicomponent systems with a minimum number of five components, the formation of at least six phases can be expected [39,40]. 

As can be seen from the concept of highly centric materials, the desired effect is the formation of simple solid solutions. The high entropy effect describes a phenomenon in which higher entropy of mixing reduces the free energy of solid solution phases and facilitates their formation, especially at higher temperatures. 

This means that the relatively low entropy of mixing observed for conventional alloys will favor the formation of chemically and physically different phases within one alloy. The type of phases formed directly affects the mechanical properties of the alloy; in the case of a solid solution, a linear change of properties as a function of temperature and thermodynamic stability can be expected. Another factor which, in addition to high entropy, reduces the number of phases formed as opposed to the Gibbs phase rule is that, unlike conventional alloys, it does not distinguish between the heavy major components. As a result, there is full mutual solubility (substitutability) between the alloying elements, which favors the formation of a smaller number of phases [33,34,35,36,37,38,39,40,41,42].

The aim of this study was to evaluate the electrochemical performance by EIS method. In order to assess the electrochemical stability as well as the resistance tendency and impedance with increasing temperatures of the charging process, Bode and Nyquist plots were examined.

## 2. Materials and Methods

### 2.1. Materials and Preparation of Carbon Material

In this research, commercial corn starch and graphene from Sigma Aldrich (USA) was used. To obtain carbon from corn starch (CSC), the dry carbonization process of materials in a tube furnace at 600 °C (increasing temperature rate 5 °C min^−1^) for 6 h with a nitrogen flow of 50 L h^−1^. The scanning electron microscope (SEM) was adopted using the EVO40 (Zeiss, Jena, Germany) apparat. 

**Figure 2:**
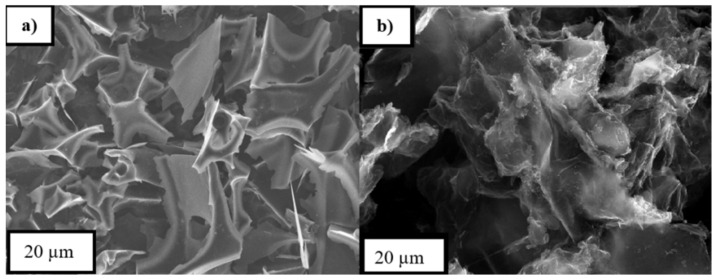
Scanning electron microscopy (SEM) images of (**a**) carbon from corn starch (CSC); (**b**) graphene in the zoom of 20 µm.

SEM images (Figure 2) show that carbon material is in the form of layers with defects, and for CSC, the layers are stiffer than for graphene, which is not that ordered and has different sizes. 

### 2.2. Electrochemical Impedance Spectroscopy (EIS)

**Figure 3:**
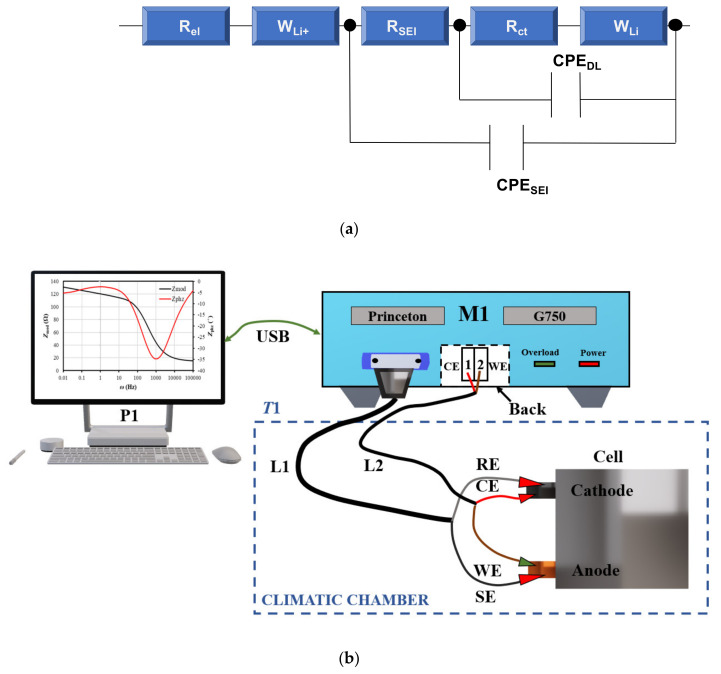
(**a**) The equivalent circuit model for impedance spectra fitting, where: *R_el_*—electrolyte resistance, *R_SEI_*—solid–electrolyte interface resistance, *R_ct_*—charge transfer resistance, *CPE*—constant phase element, *W_Li+_*—Warburg element; (**b**) potentiostat and EIS measurements, where: M1—galvanostat G750, L1—front sensor cable with shield, L2—power cable to the rear part, P1—PC with software, T1—climatic chamber.

To investigate the half-cell impedance, GTM750 Potentiostat/Galvanostat/ZRA (Gamry Instruments USA) at a frequency range of 100 kHz to 10 mHz with AC voltage amplitude equal to 10 mHz and Bode plots (*Z_mod_* and *Z_phz_* in function of frequency *ω*) were created. The measurements were carried out for increasing temperatures after the charging process. The anode paste contained polyvinylidene fluoride (PVdF)—10%, acetylene black (AB)—10%, carbonized corn starch (CSC) or graphene—80%, and N-Methyl-2-pyrrolidone (NMP). Figure 3 presents the used equivalent circuit for fitting the impedance data. The equivalent circuit for data deconvolution and potentiostat for EIS measurements are presented in Figure 3. 

## 3. Results and Discussion

A significant achievement in the technological (including electrochemical) industry is the spread of various allotropic types of carbon as industrial components. Organic electrodes require a high content of conductive carbon (over 30% by weight) to maintain proper conductivity. In order to prevent a decrease in the energy density of cells, the following are used: polymerization, salt formation, quasi-solid and completely solid electrolytes, and immobilization on solid substrates.

Cellulose, like starch, is made up of many glucose residues. The most important determinant of the applicability of cellulose is the ability to form homogeneous films or layers, as well as water solubility and ease of processing. When creating LIBs, it is not necessary to use organic solvents. CMC (carboxymethyl cellulose) and SBR-CMC (styrene-butadiene rubber–carboxymethyl cellulose) elastomer are applied, among others, showing worse electrochemical properties. Cellulose occurs naturally in the environment (mainly wood and cotton); therefore, it is characterized by low acquisition costs. Already a concentration of about 2% by weight of cellulose and its derivatives offers acceptable anode properties (300 mAh g^−1^ reversible capacity, during the first ten cycles, 20% irreversible loss).

To evaluate electrochemical stability and resistance and impedance tendencies with the increasing temperatures of the charging process, Bode and Nyquist plots were examined. Additionally, based on the Nyquist data, the activation energy of the SEI layer, electrolyte, and charge transfer processes was calculated.

### 3.1. Bode Plots

**Figure 4:**
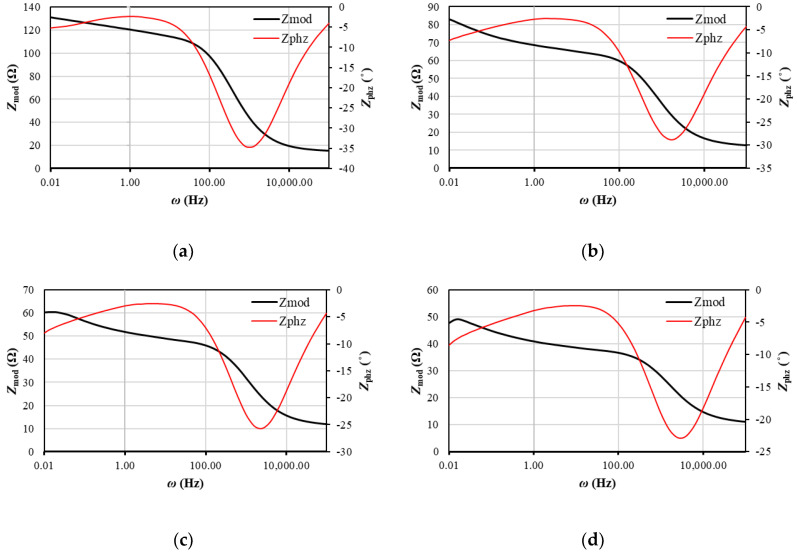

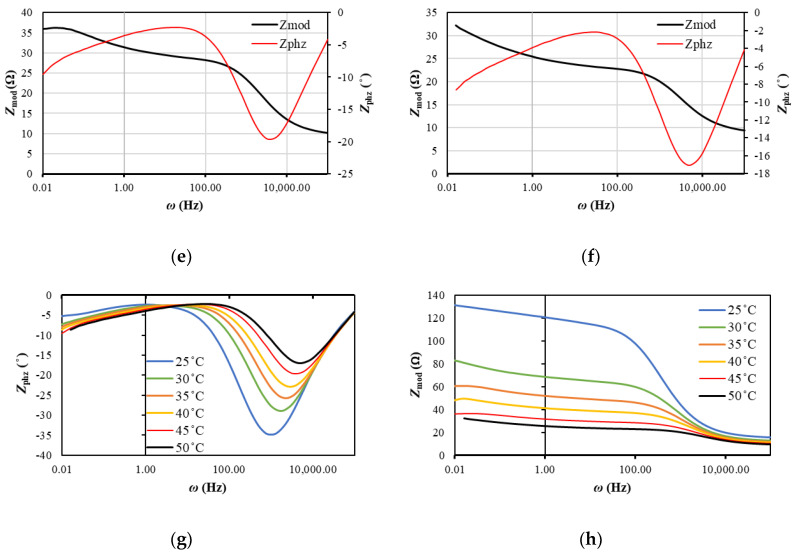
Bode magnitude and phase plot for CSC at (**a**) 25 °C; (**b**) 30 °C; (**c**) 35 °C; (**d**) 40 °C; (**e**) 45 °C; (**f**) 50 °C; (**g**) comparison of *Z_phz_* at different temperatures; (**h**) comparison of *Z_mod_* at different temperatures.

**Figure 5:**
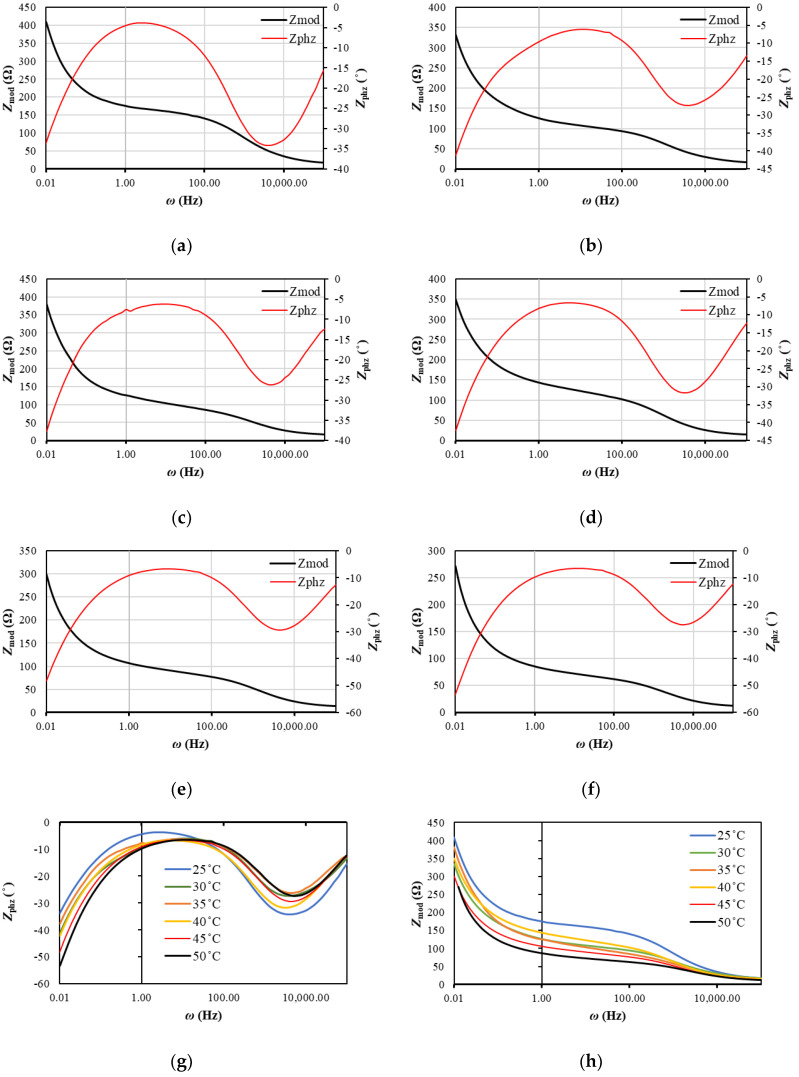
Bode magnitude and phase plot for graphene at: (**a**) 25 °C; (**b**) 30 °C; (**c**) 35 °C; (**d**) 40 °C; (**e**) 45 °C; (**f**) 50 °C; (**g**) comparison of *Z_phz_* at different temperatures; (**h**) comparison of *Z_mod_* at different temperatures.

The Bode plots in Figure 4 and Figure 5 can be used to estimate the effectiveness of lithium-ion diffusion in the electrode materials. The lithium-ion diffusion is related to the phase angle in the low-frequency region. The smaller the phase angle is, the faster the lithium-ions’ diffusion is [43]. Thus, the graphene anode exhibits a more favorable diffusion angle (−32 to −55°) compared to CSC (−5 to −10°).

According to Figure 4h, the graph can be divided into 3 regions according to frequency: (1) 100,000 Hz to 1600 Hz; (2) 1600 Hz to 10 Hz; (3) 10 Hz to 0.01 Hz.

Moreover, for graphene and CSC in the low-frequency region, the phase angles decreases as the temperature rises. The phase angle in the high-frequency region decreases significantly compared to the low-frequency region, achieving values of −17 to −33°. The opposite situation was observed for graphene, for which the phase angles have higher values in the high-frequency region than in the low-frequency region (−25 to −35°). 

At lower frequencies, the impedance (*Z_mod_*) is much higher due to mass transport effects, and at high frequencies, the impedance is mainly due to the inductance of the battery as a result of the electrodes [44]. When the temperature rises, impedances become smaller because the migration of the charges is much easier at higher temperatures. It could be seen that values of *Z_mod_* fall more drastically for CSC and achieve much smaller values than for graphene. The greatest differences between impedances are seen when the temperature increases from 25 to 30 °C. Further, the activation barrier was defeated, and the process of charge transfer in the high-frequency region and diffusion in the low-frequency region was facilitated. 

According to Figure 5h, the graph can be divided into 3 regions according to frequency: (1) 100,000 Hz to 6000 Hz; (2) 6000 Hz to 10 Hz; (3) 10 Hz to 0.01 Hz. Both for CSC (Figure 4) and for graphene, a decrease in *Z_mod_* is observed in region (1), which suggests that the electric charge is not stored by the capacitive impedance of the passive layer. The classification of the frequency regions is based on the visible changes in the nature of the curves and results from experimental studies indicating the values of resistance and impedance, and the transformation of Nyquist plots into Bode plots takes place at specific frequencies. In region (2), we also see a decline in *Z_mod_*. Region (2) connects to region (1), i.e., there is a coupled reaction of these two regions; therefore, region (2) is the border between the electrolyte and the electrode (SEI). In the case of phase angles in region (1), the difference in phase angles increases, which suggests that the passive layer at this frequency does not form in a three-dimensional form. On the other hand, in region (2), the phase angles decrease, which means that porosity of the passive layer soaked with electrolyte is observed in this area. In region (3), first an increase and then a decrease in the phase angle are observed, which means that the electrolyte initially does not cover the electrode surface evenly and then begins to fulfill this function. The greater the heterogeneity of the passive surface, the greater the ability of the three-dimensional structure to improve the diffusion of lithium ions. The smaller the phase angle, the less electrolyte soaks into the passive layer. This means that by increasing the temperature, smaller amounts of electrolyte come into contact with the electrode surface. It is also observed that the impedance and real resistances as a function of the inverse of the square of the frequency are parallel to each other and are straight (especially in the area of low frequencies), which means that the diffusion and the charge transfer reaction on the anode in the area of the tested temperatures are correct.

Bode plots for CSC look similar to the work in [35] for lithium anodes with the electrolyte: 1 M LiTFSI (lithium bis(trifluoromethanesulfonyl) imide), 1,3-dioxolan/tetraethylene glycol dimethyl ether (7:3); 1 M LiTFSI, 0.2 M LiNO_3_ (lithium nitrate), 1,3-dioxolan/tetraethylene glycol dimethyl ether (7:3); 1 M LiTFSI, PS (polysulfdes), 1,3-dioxolan/tetraethylene glycol dimethyl ether (7:3); and 1 M LiTFSI, 0.2 M LiNO_3_, PS 1,3-dioxolan/tetraethylene glycol dimethyl ether (7:3). In that case, polysulfides (PS) were found to reduce the overall oxidation state of sulfur and carbon within the SEI, while lithium nitrate (LiNO_3_) was found to increase it. Thus, it allows for the conclusion that conductive polymers decrease the general impedance of the system. Phase angle minimums for different temperatures are placed in Table 1. It could be seen, that for CSC, when the temperature rises, the phase angle achieves higher values in the higher frequency region. For graphene, two minimum phase angles in low-frequency and high-frequency region could be observed, which means that the charging process in that case is more complex.

### 3.2. Nyquist Plots

The method of interpreting the impedance spectrum for anode materials used in lithium-ion cells is presented in [45]. The correlation of the impedance spectra for the charging state turns out to be problematic due to the several-fold increase and decrease in impedance during the discharge. Only by using multivariate correlations, multiple frequencies, and numerous model parameters, a certain assessment can be achieved [46].

**Figure 6:**
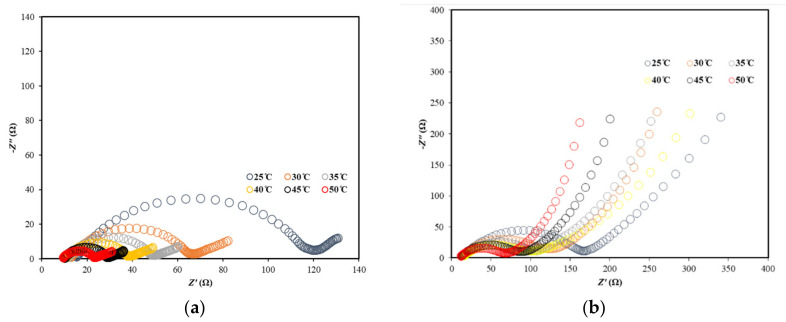
Nyquist plots for (**a**) CSC; (**b**) graphene at a temperature range of 25–50 °C.

To evaluate resistances due to different processes during the charging process, Nyquist plots were created (Figure 6a,b).

In Table 2, deconvolution data from impedance spectra (Figure 6) are contained. As the temperature rises, resistances due to SEI and the electrolyte decrease, which means that the SEI starts to decompose, and since the ion mobility increases with temperature, the resistance of the electrolyte generally falls with increasing temperatures. Electrolytic conductivity depends on two factors: ionic mobility and the degree of dissociation of the electrolyte (which increases with the increase of temperature and hence the number of ions also increases the conductivity). Thus, the energy gained by the molecules in the medium increases, and the energy state gets higher. In the case of CSC, the charge transfer is a more limiting process than for graphene anodes, where the limiting role is the Warburg part, i.e., the diffusion of lithium ions. The most complex aspect is the charge transfer of ions, the mechanism of which is connected with the capacitance of the double electrical layer. In carbon electrode materials, the main mechanism of energy storage is due to non-Faradaic double electrical layer reactions. The functional groups (e.g., hydroxyl, ether, carboxylic, anhydride, and carbonyl groups) and defects play an important role and cause pseudocapacitive effects. 

To determine the activation energy due to the resistance of the SEI layer (*E_SEI_*), electrolyte (*E_el_*), and charge transfer (*E_ct_*), the plots ln*R*^−1^ = f(*T*^−1^) were created. The slopes multiplied by the gas constant R indicate the activation energies (Arrhenius equation). The values were placed in Table 3. It can be seen that the highest differences in the activation barriers are due to the charge transfer reaction, and for graphene, this value is two times higher than for CSC. The smallest activation energy is connected with electrolyte resistance. 

A detailed analysis of the obtained material is very important due to its later use. It is known that lithium-ion batteries are widely used in the automotive industry as energy storage in hybrid and electric vehicles.

The selection of materials for their construction is key because in the case of hybrid vehicles, efforts are made to reduce the share of the combustion part of the propulsion. This is to reduce energy consumption and fuel consumption. The publication [47] presents the results of measurements of hybrid vehicles and vehicles with a range extender, which showed the effect of the degree of charging of the energy storage on the energy consumption of the drive system. In the case of vehicles with a range extender, it is a key parameter to ensure fuel consumption reduction.

Energy storage devices used in alternative drive vehicles are also recharged during the operation of the vehicle with the energy generated during the recuperation process. An example of the analysis of the balance amount of the exhaust energy recuperation process is the publication by Ziolkowski A [48]. The author presented his own design of an ATEG thermoelectric generator for automotive applications and made measurements to determine the amount of recovered electricity. At the same time, in the research on the assessment of pollutant emissions and energy consumption of various groups of machines [49,50], it becomes more and more necessary to take into account the state of charge of electrical storage devices before and after the test. The use of modern lithium-ion batteries with increased efficiency will have a positive effect on the energy balance of the vehicle, and thus will increase its environmental friendliness.

## 4. Conclusions

The resistances due to charge transfer and SEI formation (electrolyte–solid interphase) become lower after the stability test, and diffusion is the limiting process. The linear part of the graph shows the presence of the Warburg line, which results from the diffusion of lithium ions into the active material of the electrode paste. The more SEI is produced, the more stable the system becomes, as it mainly protects the electrode against corrosion and maintains good cyclical properties and increases the safety of the battery. In addition, a stable SEI is critical in attempting to achieve a high initial Coulomb efficiency. However, as the SEI grows, numerous defects form, which can lead to a significant anisotropic diffusion of the Li ions towards the electrode surface and cause non-uniform transport of Li ions to the electrode–SEI interface, resulting in lower cell efficiency.

In summary, for almost every anode system, after stabilization, the resistances resulting from the charge transfer reaction decrease, thus increasing the electrochemical intercalation capacity of lithium ions by increasing diffusion control and reducing the kinetic part. After the charge exchange semicircles, in each case, there is a Warburg curve, and lithium-ion diffusion and accumulation are the main mechanisms of the anode half-cell functioning.

Frequency characteristics are popular methods for the analysis and synthesis of linear control systems. Frequency characteristics methods are considered graphical as opposed to time characteristics methods which are directly related to differential equations and are generally based on an analytical approach.

Automatic control systems are still undergoing rapid development. This is the result of increasing requirements with regard to the control quality indicators, but also the progress in the field of new control principles (algorithms) and the development of microprocessor technology.

Bode plots for graphene and CSC anode show high electrochemical stability in increasing temperatures. The activation energy for SEI (solid–electrolyte interface) layer, charge transfer and electrolyte are in range of 24.06–25.33, 68.18–118.55, and 13.84–15.22 kJ mol^−1^, respectively. Thus, the biggest activation barrier due to intercalation process is due to charge transfer, which seems to be the most complex stage. 

## Figures and Tables

**Table 1:** Minimum phase angles for CSC and graphene with frequency approaches.

Temperature (°C)	Min. Phase Angle for CSC (°)	Min. Phase Angle for Graphene (°)
25	−34.8 (1 kHz)	−34.4 (0.013 Hz) and −34.2 (3.98 kHz)
30	−28.8 (2 kHz)	−41.2 (0.01 Hz) and −27.3 (3.98 kHz)
35	−25.7 (2.51 kHz)	−38.7 (0.026 Hz) and −26.2 (5.01 kHz)
40	−22.9 (3.16 kHz)	−42.2 (0.01 Hz) and −31.6 (3.98 kHz)
45	−19.7 (3.98 kHz)	−49.3 (0.026 Hz) and −27.4 (5.01 kHz)
50	−17.1 (5.01 kHz)	−52.4 (0.01 Hz) and −27.3 (6.31 kHz)

**Table 2:** Deconvolution data from Nyquist plots.

Parameter	*R_el_* (Ω)	*R_SEI_* (Ω)	*R_ct_* (Ω)
Sample			
G25	13.68	152.9	44.44 × 10^−1^
G30	13.37	113.30	48.81 × 10^−1^
G35	13.30	96.86	183.30 × 10^−1^
G40	12.01	89.13	1.28 × 10^2^
G45	10.20	83.46	9.17 × 10^2^
G50	8.92	66.07	8.79 × 10^2^
CSC25	15.14	58.30	1.42 × 10^−1^
CSC30	12.62	51.87	43.33 × 10^−1^
CSC35	11.50	36.28	93.11 × 10^−1^
CSC40	10.67	26.64	99.07 × 10^−1^
CSC45	9.81	18.46	135.9 × 10^−1^
CSC50	9.21	13.21	566.8 × 10^−1^

**Table 3:** Activation energies of resistances obtained during charging processes of anodes.

Parameter	CSC	Graphene
E_SEI_ (kJ mol^−1^)	25.33	24.06
E_el_ (kJ mol^−1^)	15.22	13.84
E_ct_ (kJ mol^−1^)	68.18	118.55

## Data Availability

The data are available on request.
